# Supplementary material for: A POLR3B-variant reveals a Pol III transcriptome response dependent on La protein/SSB
Source: bioRxiv. 2024 Feb 5:2024.02.05.577363. Preprint. [Version 1] doi: 10.1101/2024.02.05.577363 (PMC10896340; doi:10.1101/2024.02.05.577363)
Supplement: Supplement 1 [file NIHPP2024.02.05.577363v1-supplement-1.pdf]

SUPPLEMENTARY MATERIALS FOR

A *POLR3B*-variant reveals a Pol III transcriptome response dependent on La protein/SSB

Sandy Mattijssen[1], Kyra Kerkhofs[1], Joshi Stephen[2°], Acong Yang[3], Chen G. Han[2], Yokoyama Tadafumi[2], James R. Iben[4], Saurabh Mishra[1#], Rima M. Sakhawala[1+], Amitabh Ranjan[1], Mamatha Gowda[5], William A. Gahl[2,6], Shuo Gu[3], May C. Malicdan[2,6,*] and Richard J. Maraia[1,*]

CONTENTS

Supplementary Figures S1-S8

Supplementary Figure Legends, S1-S8

Supplementary Tables S1-S6

Supplementary Text S1

Supplementary Materials and Methods

References for Supplementary Materials

Supplementary Figure S1, Mattijssen et al

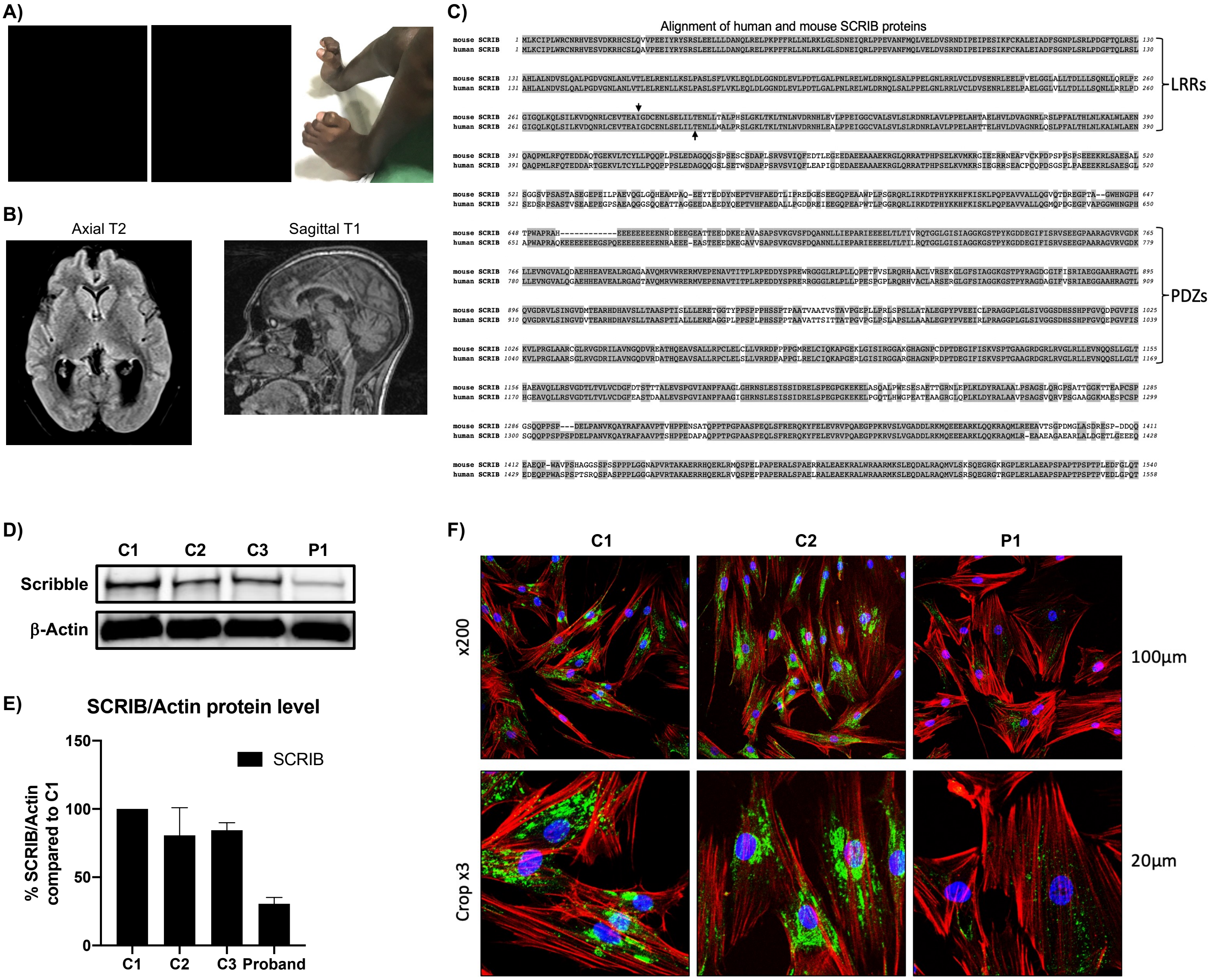

Supplementary Figure S2: Mattijssen et al.

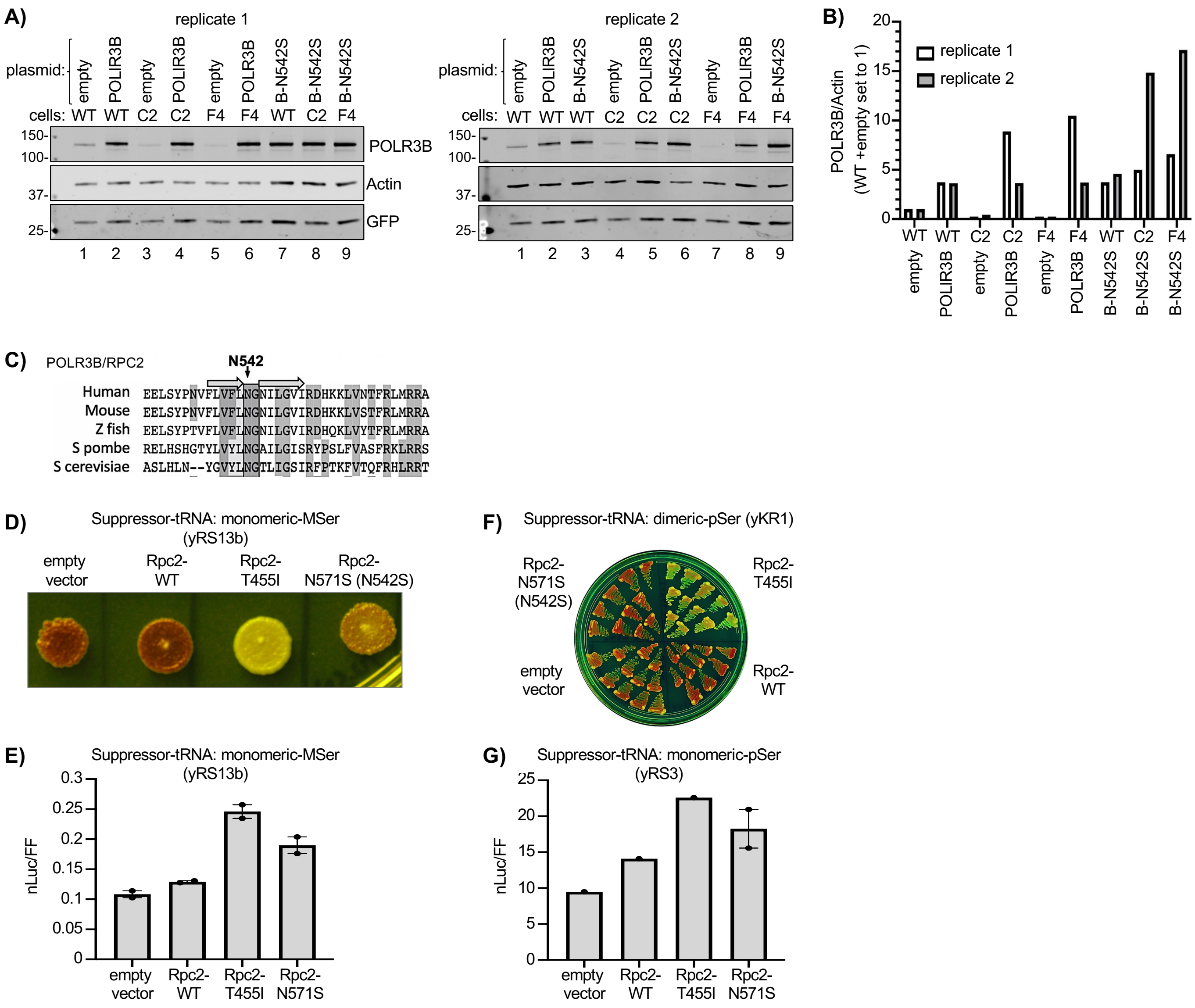

Supplementary Fig S3, Mattijssen et al.

A) 3' trailer-probed upper band (U)

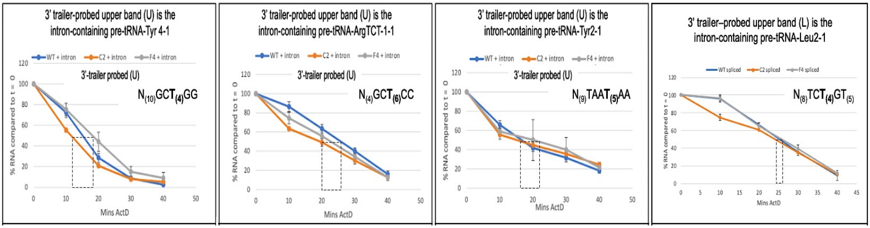

B) Intron-probed upper band

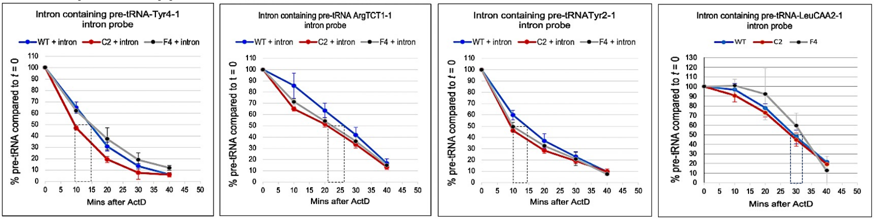

C) 3' trailer-probed lower band (L)

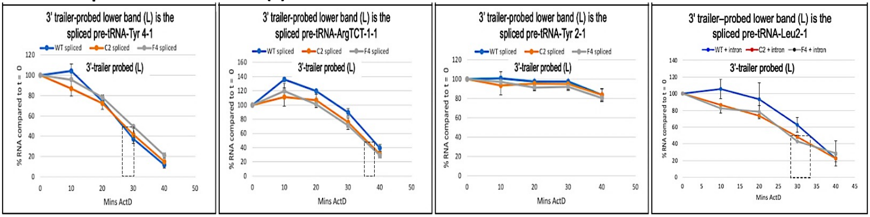

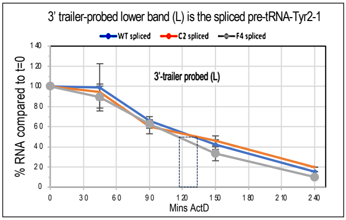

Supplementary Figure S4: Mattijssen et al.

A)

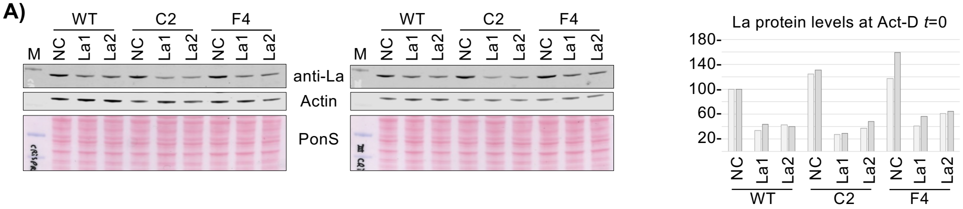

B)

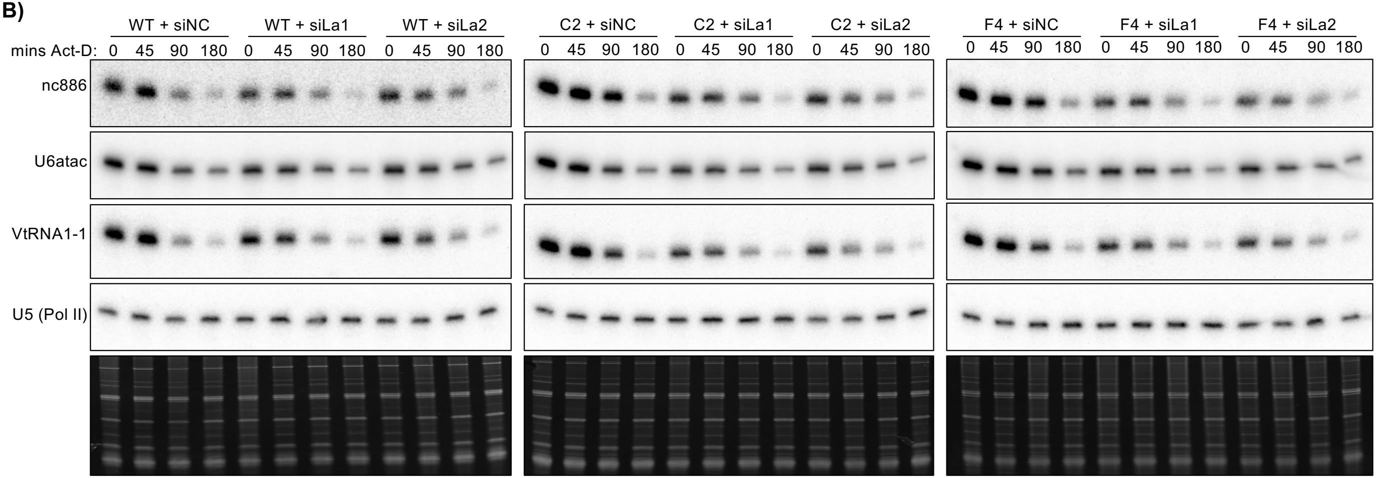

Supplementary Figure S5: Mattijssen et al.

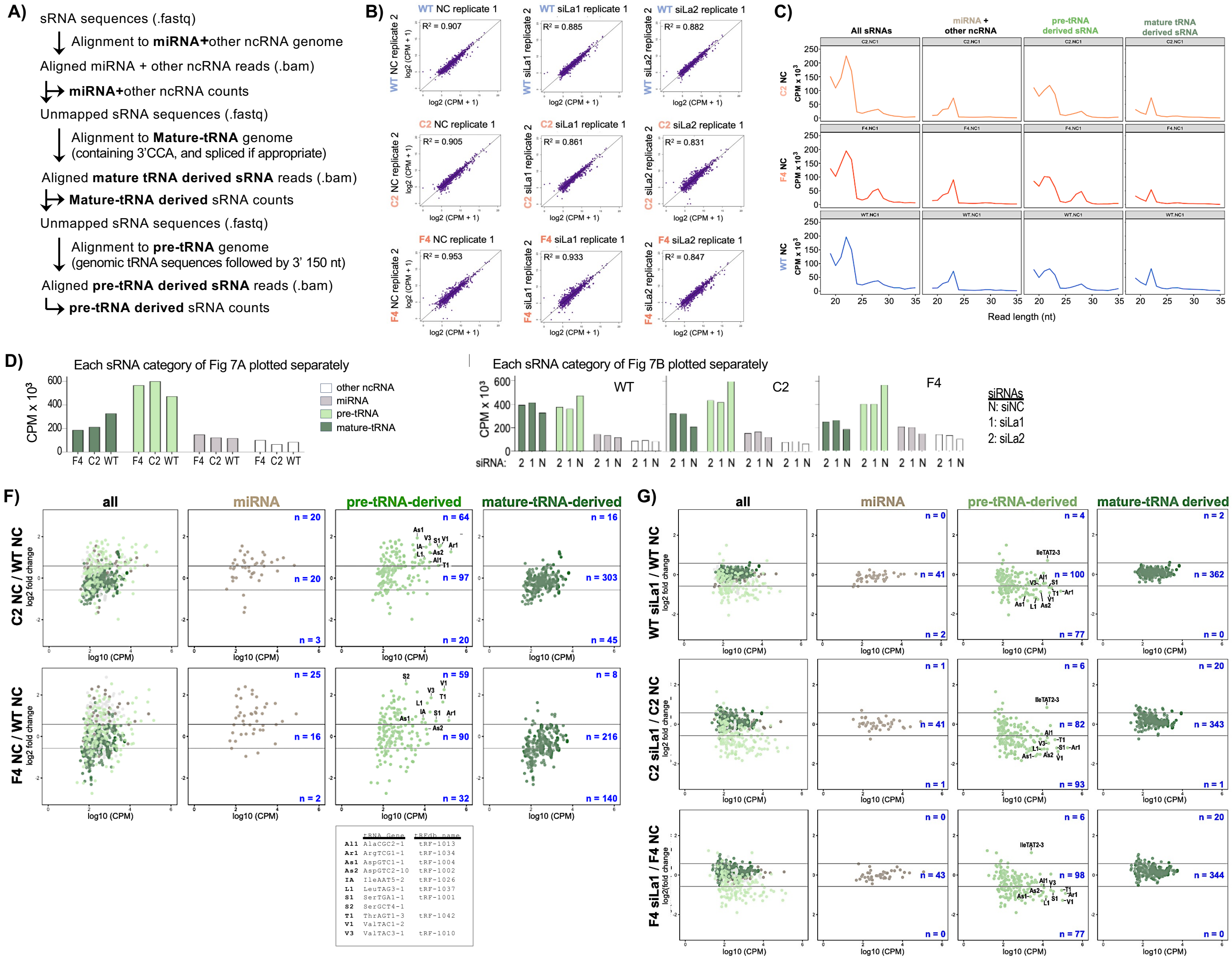

	tRNA Gene	tRFdb name
**Al1**	AlaCGC2-1	tRF-1013
**Ar1**	ArgTCG1-1	tRF-1034
**As1**	AspGTC1-1	tRF-1004
**As2**	AspGTC2-10	tRF-1002
**IA**	IleAAT5-2	tRF-1026
**L1**	LeuTAG3-1	tRF-1037
**S1**	SerTGA1-1	tRF-1001
**S2**	SerGCT4-1	
**T1**	ThrAGT1-3	tRF-1042
**V1**	ValTAC1-2	
**V3**	ValTAC3-1	tRF-1010

Supplementary Figure S6: Mattijssen et al.

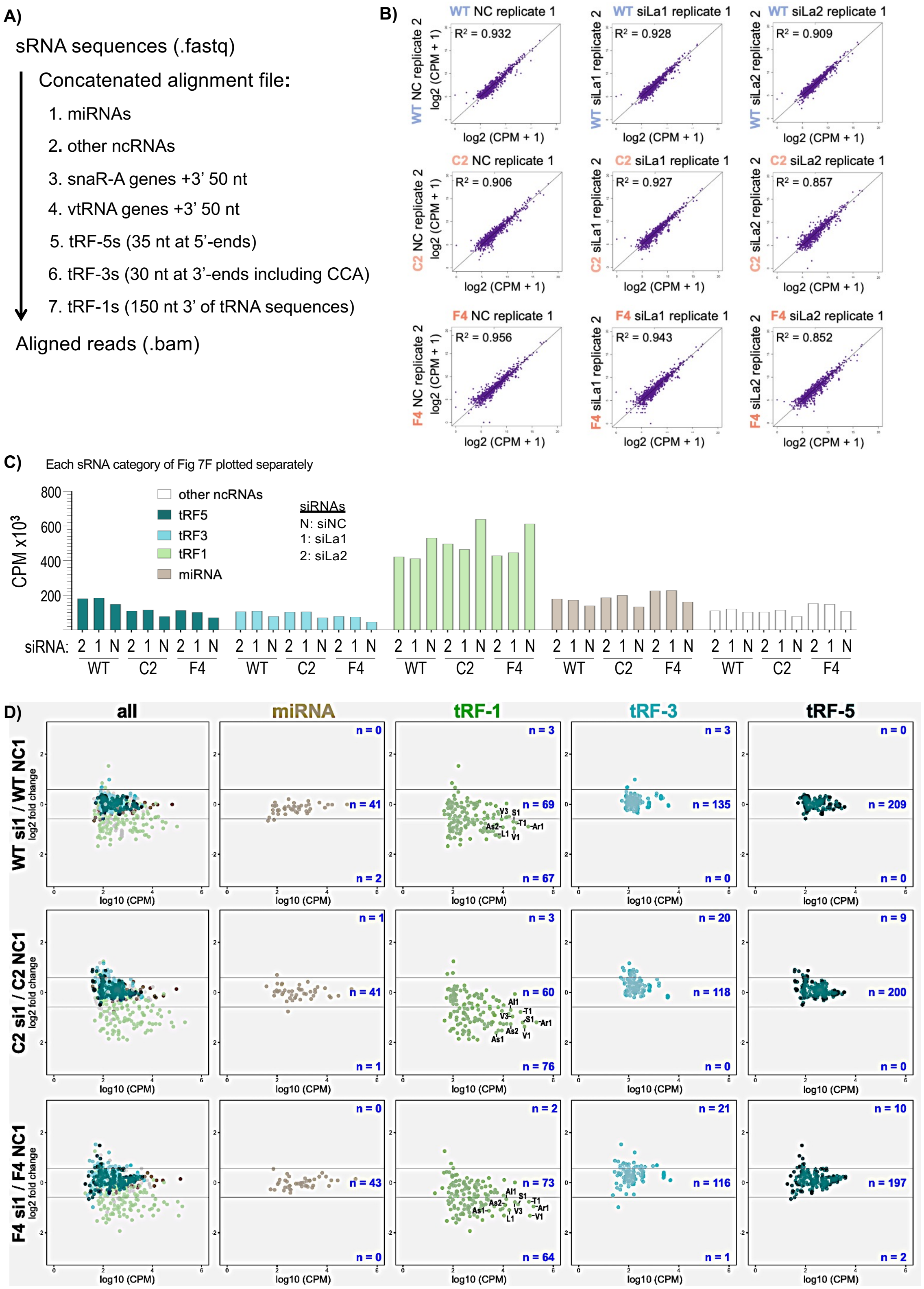

Supplementary Figure S7: Mattijssen et al.

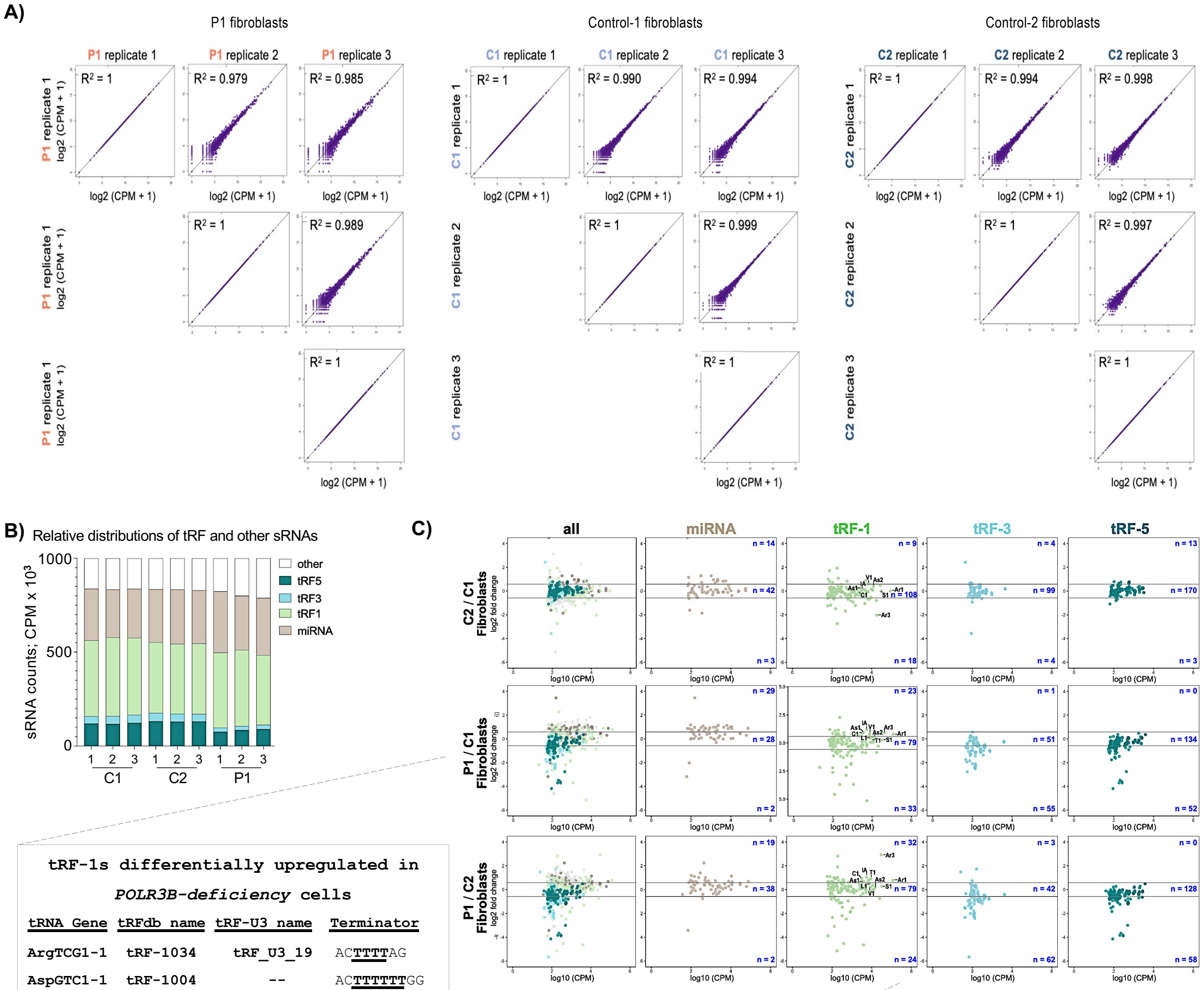

tRF-1s differentially upregulated in *POLR3B-deficiency* cells

tRNA Gene	tRFdb name	tRF-U3 name	Terminator
ArgTCG1-1	tRF-1034	tRF_U3_19	ACTTTTAG
AspGTC1-1	tRF-1004	--	ACTTTTTTGG
AspGTC2-10	tRF-1002	--	TATTTTTAA
CysGCA2-3	--	--	TGTTTTTGT
LeuTAG3-1	tRF-1037	tRF_U3_14	ACTTTTCT
ThrAGT1-3	tRF-1042	tRF_U3_7	CATTTTTGC
ValTAC1-2	--	tRF_U3_8	TGTTTTTGC
SerTGA1-1	tRF-1001	tRF_U3_1	TATTTTTTCT
IleAAT5-2	tRF-1026	tRF_U3_10	TGTTTTTTTT

	tRNA Gene	tRFdb name
Ar1	ArgTCG1-1	tRF-1034
Ar3	ArgACG1-3	tRF-1033
As1	AspGTC1-1	tRF-1004
As2	AspGTC2-10	tRF-1002
C1	CysGCA2-3	
L1	LeuTAG3-1	tRF-1037
S1	SerTGA1-1	tRF-1001
T1	ThrAGT1-3	tRF-1042
V1	ValTAC1-2	
V3	ValTAC3-1	tRF-1010

Supplementary Figure S8 Mattijssen et al

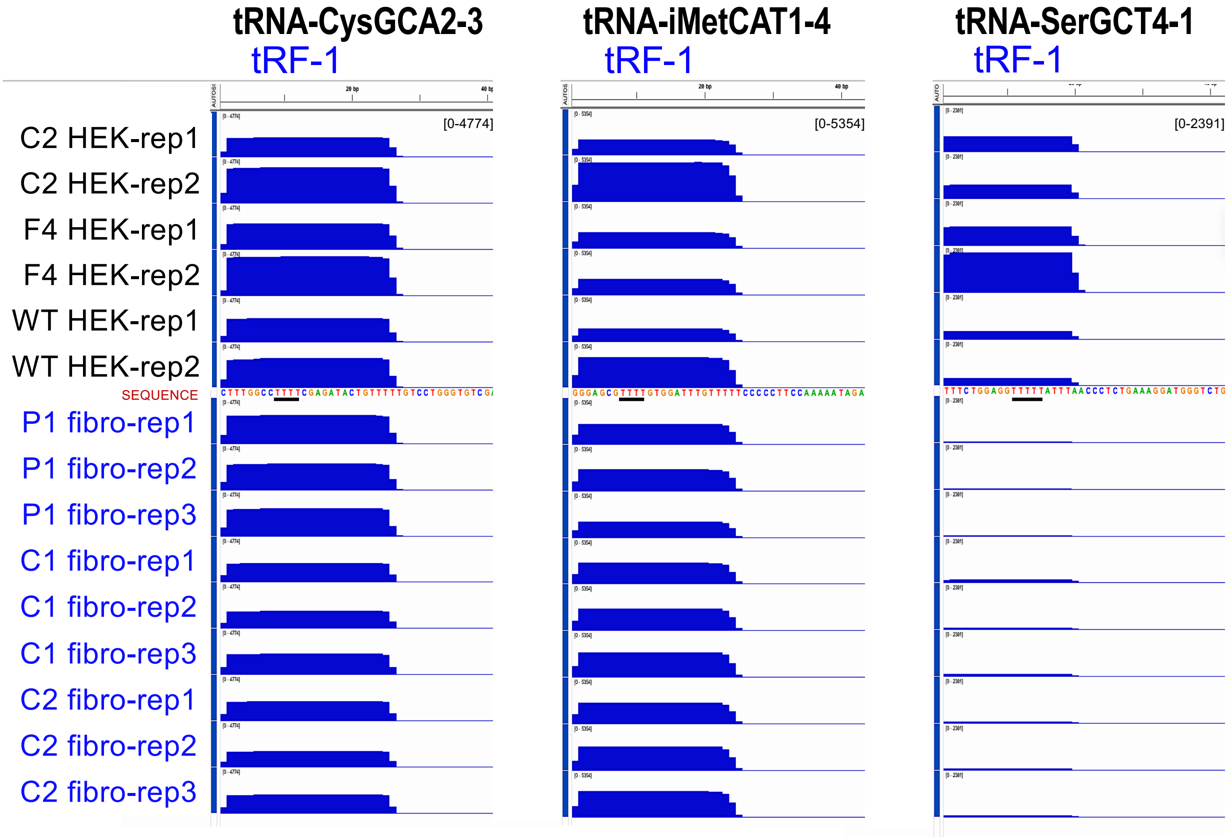

SUPPLEMENTARY FIGURE LEGENDS

Supp Fig S1: A) Images of the proband, P1 showing triangular facies, elevated nasal bridge, strabismus, hypodontia, hypotelorism, contractures of the toes and pes cavus. **B)** MRI images of P1 at 16 years of age showing colpocephaly and T2 hyperintensities only on the periventricular areas (axial T2 FLAIR image, left). Sagittal T1 image (right) shows frontal hypoplasia and mild thinning of corpus callosum. **C)** Sequence alignment of mouse and human SCRIB, a conserved protein with multiple leucine rich repeats (LRRs) that helps establish apico-basal cell polarity; mutations in *SCRIB* lead to neural tube defects in mice and human[1, reviewed in 2]. A genetic screen for developmental brain phenotypes in mice isolated multiple *SCRIB* alleles that all caused open neural tube defects in spinal cord and hindbrain; in one, the mutation was Ile285Lys[3] (down arrow) near the *SCRIB*:c.890C>G;p.Thr297Arg in the proband, P1 (up arrow). LRRs = leucine rich repeats; PDZs = PSD-95/Disc-large/ZO-1 domains. **D-F) Examination of potential pathogenicity of the *SCRIB* variant.** The *SCRIB* mutation c.890C>G; p.Thr297Arg is associated with decreased protein levels in P1 fibroblasts. **D)** Western blot of total fibroblast protein from proband (P1) and three healthy controls (C1-3). **E)** Quantitation of western blot data; C1 was set to 100%; N = 3 technical replicates (3 blots with 3 different protein amounts loaded). Actin was used for normalization; error bars represent SD. **F)** Confocal microscopy immunofluorescence. SCRIB protein is important for the polarization of epithelial and neuronal cells, and to promote actin polymerization and cytoskeletal organization. We investigated general cell morphology here but found no evidence of disorganized actin. Actin in red, SCRIB in green. Nuclei were stained with DAPI (blue).

Supp Figure S2: Western blots and quantification data for POLR3B ectopic expression duplicate rescue experiment and examination/assessment of potential effects of POLR3B-*N542S* on Pol III activity. **A)** Total protein isolated from cell lines WT, C2 or F4 transfected with empty vector (empty), FLAG-*POLR3B WT* (POLR3B) or FLAG-*POLR3B N542S* (B-N542S). The two western blots represent biological replicates. Antibodies used were against POLR3B, Actin and GFP. Actin was used as normalization control. **B)** Quantitation of the POLR3B signals on the western blots in A) using actin as a loading control as indicated on the Y-axis.

C-G) Examination of *S. pombe* Rpc2-*N571S* substitution corresponding to homologous conserved position in POLR3B-*N542S* for *in vivo* activity. To determine if the *POLR3B*-SNP might alter Pol III activity independent of mis-splicing effects, we used an *S. pombe* yeast suppressor-tRNA model system[4] for which numerous suppressor-tRNA alleles with different specific activities for suppression or designed to report on read-through past an oligo(T) terminator, are available to examine Pol III subunit and related activities[reviewed in 5]. **C)** Alignment showing that *S. pombe* Rpc2-*N571S* corresponds to conserved human POLR3B-*N542S*. **D-G)** *S. pombe* Rpc2-*N571S* was examined in three *S. pombe* strains carrying different

suppressor-tRNA alleles. **D)** yRS13b carries the MSer suppressor-tRNA with modest suppression activity. In the red-white suppression (RWS) assay, spRpc2-N571S was intermediate between Rpc2-WT and Rpc2-T455I, a dual function mutant with increased transcription and terminator read-through molecular phenotypes that can be distinguished[6,7 and refs therein]. **E)** Strain yRS13b also carries an integrated UGA stop codon-suppressible nanoluciferase ($nLuc^S$) reporter and separate firefly luciferase (FFLuc) for normalization[8]. Luciferase activity generally agreed with the RWS assay in the same strain. **F)** The increased transcription and terminator read-through molecular phenotypes of the spRpc2-T455I mutant can be distinguished[6,7 and refs therein] as terminator-readthrough can be monitored specifically by the tRNA-suppressor in strain yKR1[7]. This highly sensitive RWS assay did not detect termination deficiency by Rpc2-N571S. **G)** The yRS3 strain carries a high activity pSer suppressor-tRNA, whose levels exceed the upper limit of the RWS assay[9] which is appreciated by comparing Y-axes of panels E and G. Thus, yRS3 reports high output suppressor-tRNA gene activity (G). The cumulative data here and in HEK293 cells indicate that the missense mutation does not decrease Pol III activity. The data here suggest that when transferred to *S. pombe* Rpc2, the homologous missense substitution confers modest increase in Pol III activity.

Supp Figure S3: Graphic quantitative representation of RNA turnover from the gel blot data in Figure 4A. Triplicate time course northern blot data of total RNA at time zero and times after addition of actinomycin-D (ActD) as indicated on the X-axes. Cell lines WT, C2 and F4 are indicated by the colored lines; the tRNA gene names are indicated in the headers of each box. A, B and C, contain the RNA turnover profiles for the 3'-trailer probed upper bands (U) in panels i-iv of Fig 4A, the intron-probed band (panels vi-ix, Fig 4A) and the 3'-trailer probed lower bands (L, i-iv, Fig 4A). Vertical dashed-line rectangles extend from the points at which 50% of the RNAs remained (Y-axis), to the time in minutes on the X-axis, for estimating half-lives. Boxes in column A contain the 3'-trailer-terminator context sequences taken from Figure 4A.

Supp Figure S4: Source data corresponding to the experimental results shown in Figure 6F-G. A) Western blots. **B)** Quantitation of A. **C)** Northern blot RNA decay time course data of one of the biological duplicate experiments.

Supp Figure S5: A) Schematic overview of the sequential alignment pipeline used for small-(s)RNA analysis. Following unique molecular identifier (UMI) deduplication and adapter trimming, sRNA sequences (in .fastq format) were sequentially aligned against three custom reference genomes comprised of i) mature miRNA sequences + other ncRNAs (Methods), ii) mature intron-spliced tRNAs containing 3'-CCA, and iii) genomic pre-tRNA sequences and their 3'- 150 nucleotides (nt) downstream. Following each alignment only the unmapped reads were used to align against the next reference genome. **B)** Correlation plots of biological replicate samples of all mapped sRNAs from A after filtering low count reads; R^2 values were

calculated using the cor function in R (Methods). CPM: counts per million. **C)** Read length (X-axis) distribution of normalized reads (Y-axis). **D)** Amounts of normalized reads obtained for F4, C2, and WT cells, of each RNA type plotted separately, corresponding to Figure 7A, according to the color code. **E)** As for panel D but corresponding to Fig 7B. siRNAs referred to below are as follows; N: siNC, 1: siLa1, 2: siLa2. **F)** MA plots (Methods) of the data aligned and mapped following panel A and represented in Fig 7A-C show log2-fold (L2F) change differences (Y-axis) in levels of the sRNA types indicated above and average expression levels in log10 CPM on the X-axis; "all" represents a combination of sRNAs from the three reference genomes. The upper and lower rows show L2F changes between C2 and WT, and between F4 and WT, respectively. **G)** The same as F but the L2F changes are between cells treated with siLa1 or siNC as indicated for WT (upper row), C2 (middle) and F4 (lower). The tRNA source genes for some common abundant tRF-1s that have been validated by examination of integrated genome viewer (IGV) are indicated; the code legend is below.

Supp Figure S6: A) Schematic overview of alignment pipeline using custom reference genome set-2 for sRNA analysis (Methods). Following unique molecular identifier (UMI) deduplication and adaptor trimming, sRNA sequences (in .fastq format) were aligned to the custom genome file containing individual references according to the flow diagram and as described (Methods). **B)** Correlation plots of the biological replicate data after filtering low count reads from all RNA types according to figure 7F. **C)** Amounts of normalized reads obtained for F4, C2, and WT cells, of each RNA type plotted separately, corresponding to Figure 7F, according to the color code. siRNAs referred to below are as indicated. **D)** MA plots of log2-fold change differences on the Y-axis, and the average expression levels in log10 CPM on the X-axis for levels of miRNAs and the tRF-1s, tRF-3s and tRF-5s from individual tRNA genes, comparing each cell line treated with siLa1 or siNC as indicated for WT (upper row), C2 (middle) and F4 (lower row). The tRNA source genes for some common abundant tRF-1s that have been validated by by examination of integrated genome viewer (IGV) are indicated; for source gene annotations see legend under Supp FigS5F.

Supp Figure S7: A) Correlation plots of biological triplicate sRNA-Seq data from patient (P1) fibroblasts, control-1 (C1) and control-2 (C2) fibroblasts after filtering low count reads from all RNA types according to Supp Fig S6A. R^2 was calculated using the cor function in R. **B)** Amounts of normalized reads from P1, C1 and C2 for the different RNA types according to the color-code. **C)** MA plots of log2-fold change differences (Y-axis) and the average expression levels in log10 CPM on the X-axis for levels of miRNAs and the tRF-1s, tRF-3s and tRF-5s from individual tRNA genes, comparing C2 and C1 (upper row), P1 and C1 (middle) and P1 and C2 (lower row). The tRNA source genes for some abundant tRF-1s that have been validated by integrated genome viewer (IGV) are indicated; the legend is below. An inset-like chart lists common abundant tRF-1s that are differentially upregulated in *POLR3B-deficiency* cells (see text). Their tRFdb and U3 designations if available are provided, as well as the T1 terminator motifs of the tRNA source genes.

Supp Figure S8: IGV representations of three high expression tRF-1s that result from Pol III terminator readthrough. Duplicate samples of HEK293 *POLR3B*$^{1629A>G}$ C2 and F4, and HEK293 WT below which are triplicate samples of P1, and the two controls C1 and C2 fibroblast cells. The tRNA source genes are listed above; only the sequences downstream of the genomic tRNAs are shown, with the first ≥T4 underlined. IGV tracks were set to "group autoscale" with upper levels of the CPM ranges as follows; CysGCA2-3**:** 4774; iMetCAT1-4**:** 5354, and SerGCT4-1**:** 2391, indicated in brackets. The HEK and fibroblast sRNA-seq libraries were processed separately by the same biochemical and bioinformatic methods.

SUPP TABLE S1.
Features observed in the *POLR3B* variant proband (P1), male sibling (P2) and in POLR3-HLD/RD

Clinical feature	P1	Sibling, P2	POLR3-HLD (or -RD)
Short stature*	+	ND	++
Dental abnormalities*	+	ND	++
Developmental delay*	+	+	++
Thin corpus callosum*	+	ND	++
Hypomyelination*	-	-	++
Motor delay	+	+	++
Microcephaly	+	+	+
Colpocephaly	+	+	-
Tremor / ataxia	+	ND	++
Seizures	+	ND	+
Intellectual disability	+	ND	++
Pes cavus	+	ND	+
Increased deep tendon reflexes	+	ND	+[1]
Triangular facies	+	ND	+[2]
Vertical talus	+	ND	+[3]
Strabismus	+	ND	+[a]
Hypertelorism	+	ND	+[b]
Large ears	+	ND	+[c]
Fixed plantar flexion of toes	+	ND	-
Extension and inward rotation of foot and ankle	+	ND	-
Prominent eyes	+	ND	-
Absence of loops on thumbs and fingers	+	ND	-

ND -not determined
*Typical in POLR3-HLD[10] or in majority of cases[11]
[1]Documented in 5 patients with biallelic *POLR3A* HLD or RD[10,12] and one with biallelic *POLR1C*-HLD[13].
[2]Commonly associated with Wiedemann-Rautenstrauch syndrome (WRS) due to biallelic *POLR3A* or *POLR3G*L variants[14-18]
[3]Associated in one patient with (WRS) due to biallelic variants in *POLR3A*[16]
[a]Documented in two patients with biallelic *POLR3B*-HLD[19] and in one with biallelic *POLR1C*-HLD[20].
[b]Documented in two patients with biallelic *POLR3B*-HLD[19]
[c]Documented in one patient with biallelic *POLR3B*-HLD[19]

SUPP TABLE S2. Classification of variants according to ACMG guidelines[21].

Gene	Variant	Strong	Moderate	Supporting	Classification
SCRIB	c.890C>G (p.Thr297Arg)	-	PM2	PP3	Uncertain significance
POLR3B	c.1625A>G (p.Asn542Ser)		PM2	PP2, PP3	^Uncertain significance
POLR3B	c.1625A>G (p.Asn542Ser)	PS3	PM2	PP2, PP3	*Likely pathogenic

^Before *in vivo* functional studies. *After in vivo functionals studies were performed.
PS3: Well-established *in vitro* or *in vivo* functional studies supportive of a damaging effect on the gene or gene product.
PM2: Absent from controls or at extremely low frequency if recessive in the Exome Sequencing Project, 1000 Genomes Project, or Exome Aggregation Consortium.
PP2: Missense variant in a gene with low-rate benign missense variation and which missense is common disease mechanism.
PP3: Multiple lines of computational evidence support deleterious effect on the gene/gene product (e.g., conservation, evolutionary, splicing impact.

SUPP TABLE S3: Final candidate variants identified through exome sequencing.

Genomic position	Gene	Variant	Allele freq.	SIFT	Mutation Taster	Polyphen-2	CADD score
Chr22: 36685271C>G	*MYH9*	c.4417G>C p.Glu1473Gln	NA[#1]	[#2]Del	Disease causing	Probably damaging	27.6
Chr19: 1457191G>A	*APC2*	c.1156G>A p.Asp386Asn	NA	[#3]Tol	Disease causing	Probably damaging	29
Chr17: 56435549G>A	*RNF43*	c.1207C>T p.Pro403Ser	NA	Del	Disease causing	Probably damaging	24.8
Chr12: 106826256A>G	*POLR3B*	c.1625A>G p.Asn542Ser	0.000011	Del	Disease causing	Probably damaging	31
Chr8: 144894452G>C	*SCRIB*	c.890C>G p.Thr297Arg	0.00027	Del	Disease causing	Probably damaging	23.6
Chr13: 49030464C>T	*RB1*	c.1939C>T p.Leu647Phe	NA	Del	Disease causing	Probably damaging	27.4
Chr17: 67013895G>A	*ABCA9*	c.2803C>T p.Arg935*	0.0014	NA	NA	NA	31

[#1] NA: not applicable,
[#2]Del: deleterious,
[#3]Tol: tolerable

SUPP TABLE S4.

RNA-Seq normalized CPM data for tRNA metabolism and related factor genes

Gene name	Notes	Fold change C2 vs. WT	Fold change F4 vs. WT	C2 vs WT padj	F4 vs.WT padj
WARS1	Trp Synthetase	0.601437774	0.529077489	3.34E-51	7.58E-77
TSEN54	pre-tRNA splicing complex; molecular ruler	0.604739287	0.732257941	5.26E-21	1.17E-08
TRMT61A	Methyltransferase (MT'ase) m1A58 catalytic subunit	0.637597208	0.723686794	7.18E-10	1.69E-05
CARS2	Cys Synthetase, mitochondrial	0.64498639	0.481229545	2.22E-35	2.99E-89
TRMT5	tRNA MTase, cyto & mito tRNAs	0.645766037	0.736183377	9.71E-24	4.77E-12
MPST	Mercaptopyruvate Sulfurtransferase	0.651435535	0.535235113	5.09E-16	1.98E-31
OSGEP	t6A37, KEOPS complex	0.682602093	0.607484516	1.33E-23	1.58E-37
TRMT6	MT'ase partner, m1A58	0.685890824	0.759901308	4.11E-18	5.39E-10
GON7	t6A37, KEOPS complex	0.69226676	0.994567535	2.74E-10	0.948635921
RTRAF	Part of tRNA-splicing ligase complex	0.710493781	0.643703852	7.90E-33	1.41E-52
TRMT2A	tRNA MTase 2 Homolog A	0.712304042	0.616789483	1.93E-12	8.54E-24
ALKBH1	tRNA N1-Methyl Adenine Demethylase	0.720014999	0.827928688	6.61E-10	0.000632467
TARS2	Thr Synthetase, mitochondrial	0.733682422	0.794641942	2.36E-16	2.14E-09
ELP2	mcm^{5}U -related Elongator complex subunit	0.735920971	0.951957306	7.62E-17	0.256385241
GATB	Glutamyl-tRNA Amidotransferase Subunit B	0.744739664	0.809523797	5.38E-09	4.85E-05
RTCB	RNA 2',3'-Cyclic Phosphate And 5'-OH Ligase	0.751014577	0.691572531	6.00E-32	5.14E-51
ELP5	mcm^{5}U -related Elongator complex subunit	0.759652599	0.676809465	2.18E-09	1.14E-17
SARS2	Ser Synthetase, mitochondrial	0.776286679	0.637389933	2.36E-08	6.58E-24
NARS1	Asn synthetase	0.779990008	1.097142714	3.67E-20	0.00103155
METTL2A	tRNA N(3)-Methylcytidine MTase	0.784144411	0.830719901	2.97E-11	7.18E-07
VARS1	Val synthetase	0.785477294	0.58221073	1.25E-09	7.25E-44
QTRT1	Queuine tRNA-Ribosyltransferase Catalytic Subunit 1	0.794383413	0.785137593	3.46E-06	1.25E-06
DARS2	Asp Synthetase, mitochondrial	0.807647159	0.880575527	5.30E-10	0.000336707
XPOT	Los1; tRNA nuclear exporter	0.822641966	0.684692696	1.76E-09	2.04E-32
TP53RK	t6A37, KEOPS complex	0.83100351	0.745015889	0.000331306	1.78E-08
VARS2	Val Synthetase, mitochondrial	0.839628181	0.60071842	4.22E-06	1.35E-42
FTSJ1	FtsJ RNA 2'-O-MTase 1	0.843008739	1.065161197	5.69E-06	0.130256066
TRMT1	tRNA MTase 1	0.848302731	1.013153283	0.000672559	0.84375503
TRMT1L	tRNA MTase 1 Like	0.849715879	0.876302852	0.001644198	0.015044682
PUS7	pseudouridine synthase 7	0.856136469	1.108278181	4.07E-07	0.001060961
TSEN2	Pre-tRNA splicing endonuclease, 3' splice site	0.864577878	0.942131239	0.003187444	0.295471349
QARS1	Gln synthetase	0.867629316	0.774203421	1.11E-05	1.27E-15
IARS1	Ile synthetase	0.875905172	1.223616048	7.08E-06	4.21E-12
YARS2	Tyr Synthetase, mitochondrial	0.876167874	0.642104568	0.006556875	1.29E-19
METTL1	MTase 1, tRNA Methylguanosine	0.882134423	0.794091047	0.015309743	1.41E-05
METTL8	tRNA N3-Methylcytidine MTase, mitochondrial	0.88409504	0.840318338	0.011794659	0.000548634
XPO5	XPO5; nuclear exporter for pre-miRNAs, some tRNAs and ncRNAs	0.888680969	0.731621272	6.13E-06	5.26E-34
NSUN2	tRNA-m^{5}C; also methylates VtRNA1-1 sRNA w/ SRSF2	0.894088503	0.969022579	5.92E-09	0.165057052
QTRT2	Queuine tRNA-Ribosyltransferase Catalytic Subunit 2	0.894759098	1.227107373	0.000570623	5.79E-11
ELP6	mcm^{5}U -related Elongator complex subunit	0.899011404	0.751265318	0.007464761	1.03E-12
THUMPD1	ac^{4}C synthesis on tRNA	0.904986632	0.996270534	0.003987977	0.942968555
ADAT3	adenosine deaminase tRNA specific 3	0.908266607	0.951083578	0.330812075	0.694879511
METTL6	tRNA N(3)-Methylcytidine MTase	0.919075494	0.996563765	0.049381325	0.956235503
LARS2	Leu Synthetase, mitochondrial	0.924388066	0.845596687	0.020571444	5.52E-07
FARSA	Phe synthetase-A	0.925956941	0.938030209	0.154169286	0.283401006
IARS2	Ile Synthetase, mitochondrial	0.928358785	0.762490646	0.032419015	1.10E-16
HARS2	His Synthetase, mitochondrial	0.929411238	0.77966274	0.069546935	1.44E-10
FARSB	Phe synthetase-B	0.933759719	0.797305189	0.032988494	9.96E-14
YRDC	t6A37, KEOPS complex	0.938708876	1.153539359	0.165464347	0.000934649
C2orf49	Part of tRNA-splicing ligase complex.	0.943085237	1.234447759	0.259784031	1.01E-05
FARS2	Phe Synthetase, mitochondrial	0.948767789	0.769262968	0.397232626	1.21E-05
TRMT10A	tRNA MTase 10A	0.951881539	1.038436662	0.52844315	0.682821041
LAGE3	t6A37, KEOPS complex	0.95387406	0.726805531	0.559811064	5.06E-05
MARS2	Met Synthetase, mitochondrial	0.959514856	1.155823887	0.44341986	0.002250209
TRIT1	tRNA isopentenyltransferase 1 (i6A37)	0.960138016	1.322209784	0.432650541	7.76E-11
ELP1	mcm^{5}U, -related Elongator complex subunit	0.960160101	1.244458364	0.219822459	1.94E-15
KARS1	Lys synthetase	0.960335255	1.006786475	0.188083572	0.861138033
EARS2	Glu tRNA synthetase , mitochondrial	0.965357189	0.991336889	0.320242206	0.836343567
CTU1	Cytosolic Thiouridylase Subunit 1	0.970775122	1.018617503	0.75383899	0.873133842
TRMT10C	tRNA MTase 10C; subunit mtRNase P	0.972558791	1.085776779	0.657739658	0.143658315
TRMT61B	tRNA methyltransferase 61B; MT'ase m1A58 subunit,	0.974712108	1.375319887	0.686735959	5.97E-11
GATC	Glutamyl-tRNA Amidotransferase Subunit C	0.974917829	0.633254872	0.444453215	9.75E-73
QRSL1	Glutaminyl-TRNA Amidotransferase Subunit QRSL1	0.975409569	0.82023052	0.593868815	6.48E-08
TRMT2B	tRNA MTase 2 Homolog B	0.975524895	1.102694164	0.641326143	0.025576213
TRMT13	tRNA:M(4)X Modification Enzyme TRM13 Homolog	0.983890383	1.21161895	0.82784757	0.001126764
TRDMT1 (DNMT2)	tRNA (cytosine(38)-C(5))-MTase	0.984904535	0.928034078	0.860951955	0.354458015
THUMPD3	M2G7	0.985689161	1.001778188	0.755010582	0.970776943
TRMT10B	tRNA MTase m1A58 subunit, nuclear	0.988193038	1.692947884	0.865461904	1.28E-28
TSEN34	pre-tRNA splicing endonuclease, 5' splice site	0.992533747	0.873785367	0.911879309	0.005378072
THADA	tRNA modification	0.995130997	1.144651059	0.931923306	0.000254398
AARS2	Ala Synthetase, mitochondrial	1.007574709	0.637981031	0.885800543	6.15E-40
METTL2B	tRNA N(3)-Methylcytidine MTase	1.008285101	0.966882668	0.866920871	0.393249213
HARS1	His Synthetase	1.016732421	0.999897973	0.663382598	0.998248736
RARS1	Arg synthetase	1.020080675	0.847863542	0.686934592	7.15E-06
SARS1	Ser synthetase	1.032275486	1.065935741	0.470353891	0.102109423
TRPT1	tRNA Phosphotransferase 1; tRNA splicing 2' phosphotransferase 1	1.036480424	0.69949176	0.642692375	5.85E-07
LARS1	Leu Synthetase	1.039714718	0.950205585	0.316249235	0.170475161
WDR4	tRNA (Guanine-N7-)-MTase non-catalytic subunit WDR4	1.040423326	1.099309894	0.350413794	0.012310119
DARS1	Asp synthetase	1.042429229	1.170901185	0.19989601	1.19E-08
MARS1	Met synthetase	1.043778645	0.740188471	0.246181111	4.89E-22

PARS2	Pro Synthetase, mitochondrial	1.044812494	1.145872413	0.575318814	0.083075858
SSB	La protein	1.052985356	1.123088485	0.236946776	0.003937356
TSEN15	pre-tRNA splicing complex; interacts with TSEN34	1.057529059	1.247612152	0.309596779	1.31E-05
ALKBH8	AlkB Homolog 8, tRNA MTase	1.064251015	1.182402374	0.17623454	0.000118176
ELP3	mcm^{5}U related Elongator complex subunit	1.069114816	1.020880519	0.119542434	0.703682365
WARS2	Trp Synthetase, mitochondrial	1.082123501	1.060277853	0.105855795	0.288633857
WDR6	Trm734 homolog	1.088782718	0.845597746	0.009031014	1.50E-07
AARS1	Ala synthetase	1.088962425	0.88587727	0.004677278	5.91E-05
TRMT11	tRNA Guanosine-2'-O-MTase TRM11 Homolog	1.105790095	1.105736361	0.030779761	0.042699681
TPRKB	t6A37, KEOPS complex	1.10669619	1.165225165	0.027225532	0.001074435
EPRS1	Glu-Pro syntase	1.120556475	1.054381885	0.001261412	0.187044922
NAT10	N-acetyltransferase 10	1.130261718	1.044230997	7.99E-07	0.136534667
TRMT12	tRNA-Phe-4-demethylwyosine(37-C7) aminocarboxypropyltransferase	1.136161376	1.232580008	0.021028752	0.000224517
RARS2	Arg Synthetase, mitochondrial	1.148602094	0.804812299	4.48E-07	3.32E-14
TARS1	Thr synthetase	1.153916823	1.383194674	1.32E-08	1.26E-39
ADAT1	adenosine deaminase tRNA specific 1 (e.g., Inosine-34)	1.16988377	1.150732925	9.26E-06	0.000127281
YARS1	Tyr Synthetase	1.173565635	1.167500928	1.62E-06	4.56E-06
NARS2	Asn Synthetase, mitochondrial	1.196275436	0.960195714	1.72E-05	0.440815026
PUS3	pseudouridine synthase 4	1.222013894	1.024814588	0.000338809	0.746621322
TRMT112	tRNA Methyltransferase Activator Subunit 11-2	1.309667224	0.904155661	6.34E-08	0.065407253
ELP4	mcm^{5}U related Elongator complex subunit	1.424273229	1.118790581	2.69E-12	0.046154027
CARS1	Cys synthetase	1.435482581	1.498155332	1.57E-25	2.68E-31
GARS1	Glycine synthetase	1.534183067	1.665088685	2.15E-46	3.07E-65
CTU2	Cytosolic Thiouridylase Subunit 2	1.776621458	1.468863373	2.98E-27	2.23E-12
DALRD3	DALR anticodon binding domain containing 3	0.990479657	0.760006544	0.879726622	4.57668E-10
ELAC1 (RNase Z^S	Z^S tRNArepair/recycling; remove 2′,3′ p from ANKZF1-cleaved tRNA	0.827869736	0.850138064	0.006738911	0.028323064
ELAC2 (RNase Z^L	Z^L; pre-tRNA 3’ maturation	0.712759038	0.741423891	8.56811E-44	4.18239E-34
TRNT1	CCA-adding enzyme, also repair tRNA from Z	1.016246819	1.177394398	0.814442304	0.002032072
ANKZF1 (hydrolase)	cleaves peptidyl-tRNA in 60S-RQC between CCA-discrmnatr	0.888537971	1.142468009	0.006019157	0.002309486
CNP	CNPase participant	0.812525769	0.743214127	5.11315E-12	3.35194E-23
ANGEL2	2’/3’ cyclic phosphatase involved in pre-tRNA intron	0.894660989	0.810057536	0.06243813	0.000603302
REXO1	scRex1 homolog including pre-tRNAs	0.977556283	0.919268323	0.759233747	0.209622369
REXO2	scRex2 homolog, sc U4/U5/5S/5.8S/RNase P etc	1.47596284	1.48780571	1.23241E-21	3.85449E-22

**SUPP TABLE S5.**					
**RNA-Seq normalized CPM for Pol III and related factor genes**					
**Gene name**	Notes	**Fold change C2 vs. WT**	**Fold change C2 vs. WT**	C2 vs. WT padj	F4 vs. WT padj
	**RNA Polymerase III subunit genes**				
POLR3A	catalytic	**0.983351203**	**1.034013781**	0.659103978	0.300982402
POLR3B	catalytic	**0.350603494**	**0.260416036**	9.09E-94	1.48E-132
CRCP/R3I	Stalk heterodimer w/ POLR3H	**1.477012763**	**1.760632183**	3.54621E-27	4.61E-56
POLR3H	Stalk heterodimer w/ CRCP/POLR3I	**0.805551323**	**0.684920363**	1.73116E-09	1.34E-26
POLR3C (	Initiation heterotrimer, largest subunit	**0.981836270**	**1.276211423**	0.719670942	1.37E-11
POLR3F	Initiation trimer	**0.662888663**	**0.754729951**	4.00129E-21	2.55E-10
POLR3G	Initiation trimer alternate paralogous subunit	**1.090273912**	**1.172844469**	0.212940223	0.03091339
POLR3GL	nitiation trimer alternate paralogous subunit	**1.013204265**	**0.883386593**	0.868245479	0.063725638
POLR3D yC53	Termination-reinitiation heterodimer subunit	**0.852138845**	**0.965623591**	0.000454884	0.534717763
POLR3E yC37	Termination-reinitiation heterodimer subunit	**1.139259289**	**1.245586871**	5.75011E-06	1.30E-14
POLR3K yC11	Termination-reinitiation recycling & cleavage	**0.933750757**	**0.904282517**	0.172147365	0.049916616
POLR1C	Shared by Pols I & III	**0.944914205**	**0.845934562**	0.107831426	2.7258E-07
POLR1D	Shared by Pols I & III	**0.939147305**	**1.205701921**	0.13245686	1.14169E-06
POLR2L (core)	shared by Pols I, II & III	**1.378739280**	**1.011044730**	7.68602E-08	0.897803804
POLR2E (core)	shared by Pols I, II & III	**0.984715256**	**0.846426456**	0.824521127	0.001894249
POLR2F (core)	shared w/ Pols I, II & III	**0.876264818**	**0.891383016**	0.004230791	0.018068806
POLR2H (core)	shared w/ Pols I, II & III	**1.031413785**	**1.072372305**	0.52765249	0.108466799
POLR2K (core)	shared w/ Pols I, II & III	**1.036997450**	**0.992380559**	0.457047316	0.899296569
	**Pol III transcription factor (TF) subunit genes**				
BRF1	TFIIIB-β subunit, types-1 & 2 promoters	**0.613031875**	**0.593832546**	2.05979E-21	1.56497E-23
BRF2	TFIIIB-α subunit, type-3 promoter	0.967601611	0.931763356	0.59292784	0.23595392
BDP1	TFIIIB subunit	**1.405640742**	**1.709597138**	3.7063E-09	4.16785E-21
TBP	TFIIIB subunit, also used by Pols I and II for txn initiation	1.002699979	0.783908352	0.969437738	6.63267E-08
GTF3A	TFIIIA (5S)	0.939721285	0.866828967	0.074564017	1.76625E-05
GTF3C1	TFIIIC subunit, 220 kDa (B-Box)	0.955309198	0.908921798	0.085683334	7.95374E-05
GTF3C2	TFIIIC subunit, 110 kDa (Term),	1.075008404	0.978158003	0.007245958	0.507998456
GTF3C3	TFIIIC subunit, 102 kDa (5')	1.104843836	1.089874410	0.002192495	0.011910538
GTF3C4	TFIIIC subunit, 90 kD (bridges A-B box).	0.979078493	1.541363595	0.606115922	1.51585E-50
GTF3C5	TFIIIC subunit, 63 kDa (A-Box)	0.840906016	1.084671248	1.77154E-06	0.036711872
GTF3C6	TFIIIC subunit, 35 kDa,	0.962615250	0.721693036	0.389322145	1.7206E-18
SNAPC1	43 kDa subunit SNAPC3	0.743263732	1.204541388	9.2763E-06	0.006009813
SNAPC2	45 kDa subunit, Type-3 PSE TF subunit & Pol II PSE	0.989895075	1.117424266	0.92891482	0.30909843
SNAPC3	50 kDa subunit, Type-3 PSE TF subunit & Pol II PSE	0.990966645	1.204466706	0.87860901	3.30144E-06
SNAPC4	190 kDa subunit interacts w POU2F1	0.940878315	1.391771936	0.298539156	8.30395E-10
SNAPC5	19 kDa subunit	1.099846871	1.142488905	0.014539987	0.000727094
POU2F1	aka Oct4 coactiv w/ ZNF143/STAF	1.060771936	0.994768287	0.373232091	0.953995065
ZNF143	aka STAF, coactivator for Type-3. Binds DSE	1.188139269	1.180629837	6.65291E-05	0.000192377
**Additional regulatory genes w/ reported involvement in termination-reinitiation-recycling**					
La / SSB	Pol III nascent transcript 3'-end oligo(U) binding protein	1.061398820	1.127847803	0.23694678	0.003937356
MAF1	Pol III repressor & tumor suppressor	0.954508222	0.981660324	0.376715138	0.767053954
NFIA	Interacts with NFIB, Biotin proximity with GTF3C2 & GTF3C4	1.200407153	0.765447795	0.004930309	5.94537E-05
NFIB	Interacts with NFIX by Y2H and Biotin GTF3C2	1.026863169	0.942063269	0.690926261	0.341788866
NFIC	Interacts with TFIIIC-110	1.349775281	1.787479591	1.02701E-13	2.82934E-49
NFIX	Interacts with NFIB	1.118662761	1.256528392	0.007028596	2.89873E-08
SUB1 (PC4)		1.085220567	0.882579427	0.069573079	0.006018387
TOP1	Topoisomerase-1 Pol III termination and reinitiation; Roeder Lab	0.743155889	0.708672661	8.85908E-24	2.14622E-31
	**RNA Polymerase III regulatory factor genes**				
MYC	induces POLR3G transcription & Pol III activity independent of Max	0.798572659	0.808812586	0.000259522	0.000756345
MAX	Binds w/ Myc at Pol II genes but not at Pol III genes	0.682189291	0.741663435	1.95E-26	2.38406E-16
TP53		0.904281956	0.802126323	0.004129453	1.71001E-10
MAPK3 (ERK)		0.838228717	0.602045279	0.00019604	2.56644E-27
MAP3K3	MAP3K3: mitogen-activated protein kinase kinase kinase 3	0.845375594	0.726108581	5.19E-08	3.17038E-25
TERT	Telomerase catylc subunit	0.701311596	0.470387974	0.000107968	2.06647E-14
MAPK8 (JNK1)		1.008536466	0.988280589	0.902050683	0.862525593
RB1		1.071753479	0.997330904	0.289184061	0.975539833
CDKN2A (ARF)		0.917989288	1.285654518	0.128004602	5.07244E-06
BRCA1		0.863868153	1.015128737	5.91E-06	0.734403268
MAPK1/ERK2		0.844076952	0.711479201	1.99E-06	2.24659E-22
MAPK3/ERK1		0.838228717	0.602045279	0.00019604	2.56644E-27

SUPP TABLE S6: Oligo-DNA probes used for RNA detection. Multiple oligos for the same target were mixed together, equimolar, prior to end-labeling.

Target	Sequences 5'-3' (each line is a separate oligo, 5'-3', combined, ^{32}P-labelled and used for hybridization)	Hybridization temp., °C
POLR3A	TCGTACCCATCCTATGGTCGA<br>GCCTAGACAGTCAGCCAAGTT<br>GACTGCTCTGAAGTACCCTAC<br>TCCTCGCTTCTGAAGGTAGGT<br>ATCAGAAGTAGAGGAACATCTTCAG<br>TCAGTTTCATTGTCAGATCATCTTCATT<br>GCCAGATGAGCCATAATGCTC<br>TGTAACATAGGAATCATTGGCACAG<br>CACAGACAGCTCCTTCAGGAT<br>ACACAGGCAATCATCTGTGATATG<br>AGAGGAACTCACTCTTCTTCATGAT<br>CCTTGTCTAGCTGTGCTGTGA<br>ACGGAGCTTGGATGTGCAGAT<br>CCTCTTTCAGGAACTGCAGCA<br>CCCAGAGTTTTCTCCACCTCA	50.8
POLR3B	AAAATGCTGGAAGCAGCCTCCA<br>TTGAAGCTTTCTTCAACATCAGGAAG<br>AAGGCATTGCGGATGATCCTCT<br>CCACCTGGATCTAAGGGACATT<br>GTAGAGCTGGTAACTGAAGCTC<br>CTCTCAACACCCATGGCCTTAA<br>CCAGACTGGGCCCAAATGC<br>CTTAACTGGGACATGGGTCAGA<br>CAGAATAACTCTTCGCACCATCAC<br>AAGAGATAAAAGCTGTCCTGCCAATT<br>GCGGATATATATGACAAGCGAGAC<br>TGGCTAATTTAACAATGGGTCCATCT<br>TGTGACTGCTGGCTTCTGTTTCT<br>AAATATTCAACCAGACTCTCATGTAAGAAA<br>CTTTATCAAAAGTCTGATTGGTGTATCG	51.3
POLR3F	GCTGGGCTTCTATATGAGGCAT<br>ATAGACAACAACCTATTGATGGCTAC<br>TGCAGGTTATAGAGCATATACACCTT<br>TTCCATGGATAACTCTACCTTACTGA<br>GCCCTGTACAGTTTCATGTGTC<br>CCACTCTGTCATGTAAATACAGTTAG<br>CATGAATTAAATTATCTGCTCCCTAAGTAA<br>TTGACCCATCAGAAGTCTTGAAAGAT<br>CTCTAGCCATTATCATAGTACTTGGT<br>AGAAAATGGTGGTAGTGAACCAGATA<br>CCACACTTCATGTGATGAGGCA<br>CAAAATCCTGGTCACTGTACCAG<br>TGTGCGGAAAAGGCATGTTCCA<br>CACTTCCTGGTTCGTGGATGTT<br>TGCTGTTTCTGCCTTGGACTGT	51.3
POLR3E	GTTCAGGGTGTCGATGGC<br>CTGTAGAGTGCAGCGGCA<br>GATCTGCTTAACATCGTCTTCC<br>AGCAGGTACTGACGCTCATG<br>AGGGGCAGCGTGCGCA<br>GGTATAGGATGTCACTCTTCAC<br>GAAGTCCTTCACATCCTCGG<br>ACAAGATTATAGACTTTTTCCAGTTTGG<br>CTTTGAGGTTTTCTTCATGTTGTAAAC<br>GACTCGCCTCTTTCTAGTGGA	49.0
BRF1	GAACTGCACCTCGGACACGA<br>CAATTGCATCCTGGTAACTGGATATTT<br>CACCAAGGAGCTCCCGGTA<br>GCTTCCGACTCATTCAGGATGT<br>CCTTCTTTGCTGGCTGCGTAG<br>CCTCATCGCCATCACAGCCA<br>CAAGCACGTACACATTCACCTG<br>CTTCACAGTCCTCCTGAAGTCAT<br>GCAGTGCTGGTTCAGCTGCA<br>TCCCTCTGTTCCCGCAGGTA<br>CCGGAGCACGCTATAATTGATCTT<br>GGTCCGGTTTCCCTTGCTGA<br>TGTTCCTTGTAGATGCCGAGCT<br>GATCTTCTTCTGCTCCAGCATCTT<br>GTCATGGACACCTCGTGGTTC	51.3
HPRT1	TCGAGCAAGACGTTCAGTCC<br>GGGCTACAATGTGATGGCCT<br>TTCAGTGCTTTGATGTAATCCAGC	50.3

	GTTAAAGTTGAGAGATCATCTCCAC<br>AAAGTCTGCATTGTTTTGCCAGTG<br>CAACAAAGTCTGGCTTATATCCAAC<br>CACAAACATGATTCAAATCCCTGAAG<br>CCAAACTCAACTTGAACTCTCATC	
U6atac	ACCTTCTCTCCTTTCATACAAC<br>ATCCTTGTCAAGGGGAGTG<br>AGGTGGCAATGCCTTAACC<br>CGATGGTTAGATGCCACGA	47.5
VtRNA2-1/nc886 (specific for nc886)	CAGCACAGAGATGGACAGATAGAAAGT<br>ATGAGGAGGTAACCGCTTGAGCT	53.5
VtRNA2-1/nc886 + VtRNA1-2 (detects both)	CAGCACAGAGATGGACAGATAGAAAGT<br>ATGAGGAGGTAACCGCTTGAGCT<br>TAAGCACCCGCGGGTCTCG	53.5
VtRNA1-1	ATTAAAGAACTGTCGAAGTAACCG<br>CCAGACAGGTTGCTTGTTTC	48.2
VtRNA1-2	GGTTACAATGTACTCGAAGTAA<br>AAAAGAGCTGGAAAGCACC	45.4
VtRNA1-3	TGATGACACGCGAAGTAACCG	50.8
tRNA-Ile-TAT-1-1 intron	TGCTCCGCTCGCACTGTCA	51.8
tRNA-Tyr-GTA-2-1 intron	ATTGCCACGCCCTATCCACTACAGT	55.8
tRNA-Tyr-GTA-2-1 3'-trailer	AAATTACAGACGAAGTCCTTCGAG	50.4
tRNA-Tyr-GTA-4-1 intron	GATGTCCGCAAATGTCTATACAATCTACAG	55.4
tRNA-Tyr-GTA-4-1 3'-trailer	ATCACGCACTTCCTTCGAGCTG	56.0
tRNA-Arg-TCT-1-1 intron	GAATGCCTTCAGCCTCTAGAAG	51.3
tRNA-Arg-TCT-1-1 3'-trailer	AAAGCGTTACGACTCCGCCGG	54.7
tRNA-Leu-CAA-2-1 intron	GCAGTAAGCTTGAGTCTGGC<br>CAGAAGACCCGAACACAGGA	50.3
tRNA-Leu-CAA-2-1 3'-trailer	AAAGATCAGGCTGTGTCAGAAGTG	52.1
tRNA-Arg-TCT-4-1 5'-leader + 3'-trailer	ACAGAGACCTCACCACACACAC<br>AGAGGCACCTGCCAGGTGAC	48.0
BC200	GTTGCTTTGAGGGAAGTTACGCTT	52.1
snaR-A	GACCCATGTGGACCAGGCTG	54.4
7SL	TCCCACTACTGATCAGCACG<br>TCCCACTACTGATCAGCACG<br>AGACGGGGTCTCGCTATGTT	55.0
RMRP	TGGCGGACTTTGGAGTGGGA<br>GCGCTGAGAATGAGCCCCG	52.3
MIR (in POLRE intron)	TCTTACCCACTTAACCATACTGCC<br>TGTACAGTTTTGTTCTCGGGTCCAA<br>AAGCAGCTGTGGTGGGATTTGAA<br>CTCAGGTGGGTCACAGATGC<br>AATAGGGAGGAATACCAAACCCAGA	51.7
U5	TAAAAGGCGAAAGATTTATGCGATCTGAAGAGAAA	56.2

SUPPLEMENTARY TEXT S1; Full clinical description of proband and younger female sibling.

The proband (P1) was a 22-year-old male (Fig 1A) from a consanguineous family, with prenatal ultrasound detection of microcephaly and a clinical history of neurological disease and developmental delay. He was a product of full-term birth, normal delivery who cried at birth. when seen in the clinic he had severe microcephaly (<1st percentile, OFC 38 cm) and motor developmental delay. He was unable to stand without support nor stand up from sitting position nor had achieved any verbal milestones. He had attention deficit hyperactive disorder, drooling of saliva and seizures from two years of age controlled by antiepileptic medication. Other features included short stature (108 cm), triangular facies, elevated nasal bridge, hypotelorism, strabismus, hypodontia, contractures of toes, pes cavus and complete absence of loops in thumbs and fingers (Fig 1B). The patient passed away in 2022 at ~24 years old.

Hearing and vision were normal at testing. CT scan showed microcephaly, a small frontal lobe with few sulci, and colpocephaly. MRI performed at age 16 years showed bilateral frontal hypoplasia, colpocephaly, moderate thinning of the corpus callosum, and completed myelination (Fig 1C).

The younger male sibling (P2) expired at age 10 with a history of a more severe phenotype. Antenatal USG showed microcephaly. CT showed microcephaly, small frontal lobe with few sulci, bifrontal subdural hygroma, colpocephaly, few calcifications around the left temporal lope and dilated lateral ventricles.

SUPPLEMENTARY MATERIALS AND METHODS

Final sequence candidate variant filtering. Five of the seven genes found with homozygous variants by exome analysis were excluded after review of available literature as summarized here. *ABCA9* encodes an ATP-binding cassette (ABC) transporter involved with translocation of various substrates across membranes that is poorly expressed in nervous system tissues. *ABCA9* variants are not associated with human disease; deletion of this gene in mice leads to reduced anxiety. *RB1* (retinoblastoma transcriptional corepressor-1) is a tumor suppressor gene. Somatic mutations in *RB1* are associated with various specific types of cancer (MIMs 180200, 109800, 259500 and 182280, none of which were detected in the proband. *MYH9* codes for non-muscle myosin heavy chain that contributes to cytoskeleton reorganization. Individuals with variants in *MYH9* have autosomal dominant deafness (MIM 603622) that can be associated with macrothrombocytopenia and granulocyte inclusions (MIM 155100). Our proband does not present with deafness. Variants in *APC2* are associated with Sotos syndrome (MIM 617169) and complex cortical dysplasia (MIM 618677), neither of which is a phenotypic match with our proband. Nonsense heterozygous germline variants in *RNF43* have been implicated in polyposis cancer syndrome (MIM 617108), while truncating or inactivating variants were identified in colorectal adenocarcinomas. No neurologic symptoms were detected in adults with *RNF43* variants.

Immunofluorescence/immunostaining of SCRIB protein was as described[22] using primary anti-SCRIB, rabbit polyclonal (Sigma, HPA023557) at 1:200 and Alexafluor 488 conjugated Goat anti-Rabbit (ThermoFisher) as secondary antibody. Phalloidin was used for actin staining. Images were visualized and captured on a Zeiss LSM700 confocal laser-scanning microscope using a 20X objective and analyzed using Zen black 2012 LSM software (Carl Zeiss Microscopy GmbH, Jena, Germany).

tRNA mediated suppression (TMS) in *S. pombe*. The pRep4X plasmid-mediated expression in *S. pombe* harboring *ade6-704* and integrated suppressor-tRNA genes was as described[5]. *S. pombe* Rpc2-WT and Rpc2-T455I were published[6,7]; Rpc2-N571S was made by site-directed mutagenesis using Q5 Site-Directed Mutagenesis (NEB) and primers: 5'-GGTTTATTTATCTGGTGCTATTTTAGGTATTAGC and 5'AAGTAGGTACCATGGCTG. All constructs were verified by sequencing.

The Mser and pSer suppressor tRNA alleles exhibit intrinsically different specific activities for suppression, independent of terminator length. Strain yRS13b (h-*ade6-704* ura4-D18 *leu1*-32:tRNAmSer5T-*leu1*$^{+}$ nLuc$^{\mathbf{S1}}$Kan$^{\mathbf{R}}$FLuc) was transformed with pRep4X containing Rpc2, derivatives or empty vector and plated on minimal media lacking uracil with adenine at 10 mg/L. Representative transformant colonies were spotted onto the same plates and grown at 32°C for color development. The same transformant colonies used for the above red-white spotting assay were grown overnight in liquid media lacking uracil, diluted then grown to OD_{600} of 0.5 in 25 ml media. Cells were harvested, and lysates

were made into Glo buffer (Promega). Nano-Glo Dual-Luciferase assays were carried out according to the instructions (Promega). The same was done for yRS6 (h-*ade6-704* ura4-D18 *leu1*-32:tRNApSer7T-*leu1*$^{+}$ nLuc$^{\mathbf{S1}}$Kan$^{\mathbf{R}}$FLuc). The nLuc$^{\mathbf{S1}}$Kan$^{\mathbf{R}}$FLuc inserted on *S. pombe* chromosome I was described[8]; the suppressor tRNA gene is at the *leu1* locus[5]. The nLuc$^{\mathbf{S1}}$ indicates it is opal-suppressible, with ser-TGA codon-39 recognized by suppressor-tRNA *sup3-e* and derivatives[5]. The yKR1 strain was described[7].

REFERENCES for Supplementary Materials

1. Robinson, A. et al. Mutations in the planar cell polarity genes CELSR1 and SCRIB are associated with the severe neural tube defect craniorachischisis. *Hum Mutat* **33**, 440-7 (2012).
2. How, J.Y., Stephens, R., Lim, K.Y.B., Humbert, P.O. & Kvansakul, M. Structural basis of the human Scribble-Vangl2 association in health and disease. *Biochem J* **BCJ20200816.**(2021).
3. Zarbalis, K. et al. A focused and efficient genetic screening strategy in the mouse: identification of mutations that disrupt cortical development. *PLoS Biol* **2**, E219 (2004).
4. Arimbasseri, A.G. et al. RNA polymerase III output is functionally linked to tRNA dimethyl-G26 modification. *PLoS Genetics* **11**, e1005671 (2015).
5. Rijal, K., Maraia, RJ & Arimbasseri, AG. A methods review on use of nonsense suppression to study 3' end formation and other aspects of tRNA biogenesis. *Gene* **556**, 35-50 (2015).
6. Iben, J.R. et al. Point mutations in the Rpb9-homologous domain of Rpc11 that impair transcription termination by RNA polymerase III. *Nucleic Acids Res* **39**, 6100-6113 (2011).
7. Rijal, K. & Maraia, R.J. Active Center Control of Termination by RNA Polymerase III and tRNA Gene Transcription Levels In Vivo. *PLoS Genet* **12**, e1006253 (2016).
8. Mishra, S., Hasan, S.H., Sakhawala, R.M., Chaudhry, S. & Maraia, R.J. Mechanism of RNA Polymerase III termination-associated reinitiation-recycling conferred by the essential function of the N terminal-and-Linker domain of the C11 subunit. *Nat Commun* **12**, 5900 (2021).
9. Arimbasseri, A.G., Kassavetis, G.A. & Maraia, R.J. Comment on "Mechanism of eukaryotic RNA polymerase III transcription termination". *Science* **345**, 524 (2014).
10. Perrier, S. et al. Expanding the phenotypic and molecular spectrum of RNA polymerase III-related leukodystrophy. *Neurol Genet* **6**, e425 (2020).
11. Wolf, N.I. et al. Clinical spectrum of 4H leukodystrophy caused by POLR3A and POLR3B mutations. *Neurology* **83**, 1898-905 (2014).
12. de Assis Pereira Matos, P.C.A. et al. POLR3A-Related Disorder Presenting with Late-Onset Dystonia and Spastic Paraplegia. *Mov Disord Clin Pract* **7**, 467-469 (2020).
13. Kashiki, H. et al. POLR1C variants dysregulate splicing and cause hypomyelinating leukodystrophy. *Neurol Genet* **6**, e524 (2020).
14. Báez-Becerra, C.T. et al. Nucleolar disruption, activation of P53 and premature senescence in POLR3A-mutated Wiedemann-Rautenstrauch syndrome fibroblasts. *Mech Ageing Dev* **192**, 111360 (2020).
15. Beauregard-Lacroix, E. et al. A variant of neonatal progeroid syndrome, or Wiedemann-Rautenstrauch syndrome, is associated with a nonsense variant in POLR3GL. *Eur J Hum Genet* (2020).
16. Jay, A.M. et al. Neonatal progeriod syndrome associated with biallelic truncating variants in POLR3A. *Am J Med Genet A* **170**, 3343-3346 (2016).
17. Paolacci, S. et al. Specific combinations of biallelic POLR3A variants cause Wiedemann-Rautenstrauch syndrome. *J Med Genet* **55**, 837-846 (2018).
18. Wambach, J.A. et al. Bi-allelic POLR3A Loss-of-Function Variants Cause Autosomal-Recessive Wiedemann-Rautenstrauch Syndrome. *Am J Hum Genet* **103**, 968-975 (2018).
19. Kulhánek, J. et al. POLR3B-associated leukodystrophy: clinical, neuroimaging and molecular-genetic analyses in four patients: clinical heterogeneity and novel mutations in POLR3B gene. *Neurol Neurochir Pol* **53**, 369-376 (2019).
20. Gauquelin, L. et al. Clinical spectrum of POLR3-related leukodystrophy caused by biallelic POLR1C pathogenic variants. *Neurol Genet* **5**, e369 (2019).
21. Richards, S. et al. Standards and guidelines for the interpretation of sequence variants: a joint consensus recommendation of the American College of Medical Genetics and Genomics and the Association for Molecular Pathology. *Genet Med* **17**, 405-24 (2015).
22. Stephen, J. et al. Cellular and molecular defects in a patient with Hermansky-Pudlak syndrome type 5. *PLoS One* **12**, e0173682 (2017).
